# Influence of global warming and human activity on mercury accumulation patterns in wetlands across the Qinghai–Tibet Plateau

**DOI:** 10.1093/nsr/nwae414

**Published:** 2024-11-15

**Authors:** Xinbin Feng, Xun Wang, Longyu Jia, Wei Yuan, Meng Lu, Nantao Liu, Fei Wu, Xinyuan Cai, Feiyue Wang, Che-Jen Lin

**Affiliations:** State Key Laboratory of Environmental Geochemistry, Institute of Geochemistry, Chinese Academy of Sciences, Guiyang 550081, China; University of Chinese Academy of Sciences, Beijing 100049, China; State Key Laboratory of Environmental Geochemistry, Institute of Geochemistry, Chinese Academy of Sciences, Guiyang 550081, China; State Key Laboratory of Environmental Geochemistry, Institute of Geochemistry, Chinese Academy of Sciences, Guiyang 550081, China; University of Chinese Academy of Sciences, Beijing 100049, China; State Key Laboratory of Environmental Geochemistry, Institute of Geochemistry, Chinese Academy of Sciences, Guiyang 550081, China; School of Ecology and Environmental Science, Yunnan University, Kunming 650091, China; State Key Laboratory of Environmental Geochemistry, Institute of Geochemistry, Chinese Academy of Sciences, Guiyang 550081, China; State Key Laboratory of Environmental Geochemistry, Institute of Geochemistry, Chinese Academy of Sciences, Guiyang 550081, China; University of Chinese Academy of Sciences, Beijing 100049, China; State Key Laboratory of Environmental Geochemistry, Institute of Geochemistry, Chinese Academy of Sciences, Guiyang 550081, China; University of Chinese Academy of Sciences, Beijing 100049, China; Centre for Earth Observation Science, and Department of Environment and Geography, University of Manitoba, Winnipeg, MB R3T 2N2, Canada; Center for Advances in Water and Air Quality, Lamar University, Beaumont, TX 77710, USA

**Keywords:** mercury pollution, wetland, Tibetan Plateau, isotopes, sediment

## Abstract

Wetlands in the Qinghai–Tibet Plateau are a unique and fragile ecosystem undergoing rapid changes. We show two unique patterns of mercury (Hg) accumulation in wetland sediments. One is the ‘surface peak’ in monsoon-controlled regions and the other is the ‘subsurface peak’ in westerly-controlled regions. The former is attributed to the combined effects of increasing anthropogenic emissions and climate-induced changes in the cryosphere and wetland hydrology in the last 100−150 years. The climate changes in westerly-controlled regions in the last 50−70 years led to a fluctuation in hydrology and Hg peak in the sediment subsurface. The increase in legacy Hg input from soil erosion has largely enhanced the Hg accumulation rate in wetlands since the 1950s, especially in the proglacial wetlands. We highlight that accelerated glacier melting and permafrost thawing caused by global warming have altered geomorphology and hydrology, and affected Hg transport and accumulation in wetlands.

## Teaser

Glacier melting and permafrost thawing forced by global warming, coupled with human influences, affect Hg transport and accumulation patterns in wetlands of the Qinghai-Tibet Plateau.

## INTRODUCTION

Mercury (Hg) is one of the most problematic pollutants of global concern. It has directly or indirectly affected the health of millions of people and cost billions of dollars in damage [1–3]. Wetlands are transition zones where the flow of water, the cycling of nutrients and solar energy meet to produce an ecosystem characterized by a unique dynamic of hydrology, soils and vegetation [4]. Wetlands are known to be a Hg-sensitive ecosystem due to the anoxic biochemical production of methyl Hg (MeHg)—a potent neurotoxin that biomagnifies in aquatic food webs and poses a threat to human health [5–9]. Understanding Hg cycling in wetlands is crucial for evaluating the effectiveness of the Minamata Convention on Mercury—a legally binding international treaty that protects human health and the environment from the adverse effects of anthropogenic Hg emissions.

Known as the ‘roof of the world’ and ‘water tower of Asia’, the Qinghai–Tibet Plateau (QTP) is a key eco-safety barrier, and a focus of China and the region to promote ecological sustainability. With an average altitude of >4000 m above sea level, the QTP is a remote ecosystem that is sensitive to anthropogenic impacts from the world's highest Hg emission region (i.e. South and East Asia) [10,11]. The QTP has also been experiencing rapid climate warming, cryosphere melting and intensified human activities [12,13]. These changes interact with the Hg cycle by forcing physical, biogeochemical and ecological factors that control the Hg pollution risk in the QTP [14,15]. Anthropogenic Hg emissions have had an increased Hg accumulation rate in lake sediments and ice cores of the QTP by two to seven times since the 1850s [16–18]. Global warming further accelerates Hg accumulation in lakes by releasing legacy Hg from melting glaciers [19].

Gaseous elemental Hg (Hg^0^), which is the dominant form of Hg emissions from human activities, can be transported to the QTP region and deposited into wetlands following Hg^0^ oxidation to Hg^2+^ (i.e. atmospheric Hg^2+^ deposition) [16,17]. The uptake of atmospheric Hg^0^ by vegetation and its subsequent deposition through litterfall (i.e. atmospheric Hg^0^ deposition) represents another atmospheric source of Hg input to wetlands [20,21]. Other Hg sources include geogenic Hg (e.g. rock weathering) and Hg release from the melting glaciers and thawing permafrost of the region. The interplay of these sources in a changing climate and their impacts on the translocation and accumulation in wetlands of the QTP remain poorly studied.

Recent advancements in Hg stable isotope analysis have enabled the investigation of such complex natural processes. Stable Hg isotopes exhibit mass-dependent fractionation (MDF, expressed as δ^202^Hg), odd mass-independent fractionation (odd-MIF, Δ^199^Hg and Δ^201^Hg) and even mass-independent fractionation (even-MIF, Δ^200^Hg). Each of the fractionations is indicative of specific biogeochemical processes [22,23]. The Hg isotope is a powerful tool for identifying Hg accumulation processes and sources in the QTP wetlands. This can be further supported by analyses of ^210^Pb and ^14^C to determine the age of the sediment and the turnover of organic carbon in wetlands [16,24,25].

Therefore, the objectives of this study are to quantify the spatial distribution of Hg accumulation in wetlands of the QTP, understand Hg sources and accumulation processes, and identify the impact caused by climate change and long-distance transportation of anthropogenic Hg release. We determined the Hg vertical distributions in 50 sediment cores retrieved from wetlands across the QTP, and applied the stable isotopic and chronological signatures to determine the variations in Hg accumulation and sources. The implications of increasing Hg accumulation in the QTP wetlands in a warming climate for the Minamata Convention on Mercury were also discussed.

## RESULTS AND DISCUSSION

### Spatial distribution of Hg concentration

**Figure 1.**
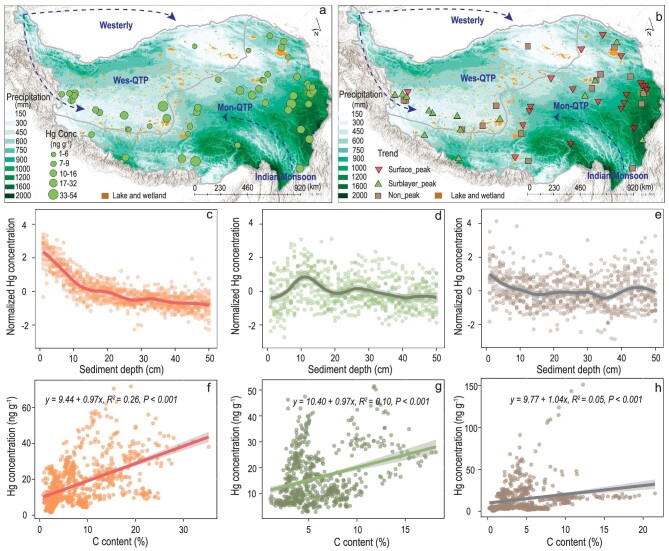
Distribution of the Hg concentration and its correlations with various parameters in the top 50-cm cores of wetland sediments across the QTP. (a) Map showing average Hg concentrations and annual precipitation amounts; (b) map showing the types of vertical Hg trend; (c) Hg concentration decreasing with the depth of cores (surface peak sites); (d) Hg concentration peaking in the sub-layer of the core (subsurface peak sites); (e) Hg concentration with a non-consistent trend (non-consistent peak sites); (f–h) Hg concentration versus carbon (C) content at surface peak, subsurface peak and non-consistent peak sites, respectively. The average precipitation data for 2010–2020 are from the Tibetan Plateau Science Data Center (http://data.tpdc.ac.cn). The normalized Hg concentration means the Hg concentration is normalized by the mean value and standard deviation in each sediment core.

The mean Hg concentration in the top 50-cm depth of 50 sediment cores ranges from 1.4 ± 0.6 to 53.7 ± 30.9 ng g^−1^ (mean ± standard deviation; Fig. 1a). Mercury concentrations are comparable between lacustrine wetlands, riverine wetlands and marshes (Figs S1 and S2). To explore the spatial distribution, we divided wetlands into two groups based on the climate zone—namely westerly- and monsoon-controlled regions (Wes- and Mon-QTP, respectively). The Mon-QTP has higher annual precipitation (>400 mm) and greater vegetation biomass than the Wes-QTP [26]. The Mon-QTP has a higher average sediment Hg concentration than the Wes-QTP (16.7 versus 13.7 ng g^−1^, *P* < 0.001, Fig. S3). Higher Hg concentrations in the Mon-QTP can be attributed to two main causes. One is that precipitation plays an important role in shaping the spatial Hg accumulation in sediments because higher precipitation enhances both the wet deposition of atmospheric Hg^2+^ and atmospheric Hg^0^ deposition due to enhanced vegetation biomass production [27,28]. Another is that the long-distance transportation of anthropogenic Hg emission contributes to the increasing trend of Hg in the Mon-QTP. The Mon-QTP is influenced more significantly by the Indian monsoon [13,29], which is known to transport anthropogenic Hg emissions from India, Southeast Asia and Southeast China [14,15]. The altitude span of ≤5000 m from the South Asia Plain to the Himalaya effectively hinders the atmospheric trans-boundary transportation of Hg [17], while the altitude span of 1000−2000 m from Southwest China and South/Southeast Asia to the Mon-QTP leads to a much easier atmospheric transport pathway.

More than 50% of the sites (19 of 35) in the Mon-QTP show a surface peak Hg concentration within the top 20 cm of the sediment depth (Fig. 1b and c). In contrast, 60% of the sites (9 of 15) in the Wes-QTP show a subsurface Hg concentration peak at intermediate depths of 10−30 cm in the sediment (Fig. 1b and d). Sixteen sites do not show a clear vertical trend in Hg concentrations (Fig. 1b and e). Such variations in the vertical distribution pattern indicate complex biogeochemical processes affecting Hg accumulation in sediments (e.g. various Hg sources mixing, impacts from local climate and geographical heterogeneities, etc.). We selected 17 sediment cores with >5% total C content for further examination because the C-rich sediment cores have relatively fewer geogenic impacts [20]. The Hg concentration in these cores mostly shows a distinct surface peak in the Mon-QTP and a subsurface peak in the Wes-QTP (Fig. S4).

The cycling of Hg links closely to that of carbon because of vegetation assimilation of atmospheric Hg^0^, Hg complexation by organic matter and organic-matter-mediated Hg transformation in soil [27,28]. The Hg concentration shows the strongest correlation with carbon at surface peak sites, then at subsurface peak sites and the weakest correlation at non-consistent peak sites (Fig. 1f, g and h). This gradient of correlation coefficients can be attributed to the higher environmental disturbances (e.g. changes in hydrology, vegetation and climate) that reduce the relations of Hg and C at the subsurface peak sites.

The vertical Hg trends in earlier natural archives (i.e. lake sediment, peat and ice cores) usually showed a slow Hg increase during the 1850s to 1950s and a rapid increase since the 1950s [16,18,30–33]. This is because of the rapid increase in global anthropogenic Hg emissions since the 1950s, specifically from the 1980s, and the increasing anthropogenic Hg emissions in Asia [11,34]. To depict Hg vertical accumulation patterns in the QTP, we selected Core DS1, DS2 and DS3 (decreasing downwards) in the Mon-QTP and DS4 (Hg peak at the intermediate depths of 10–15 cm) and DS5 (Hg peak at the intermediate depths of 30–35 cm) in the Wes-QTP for chronological dating (Fig. S4).

**Figure 2.**
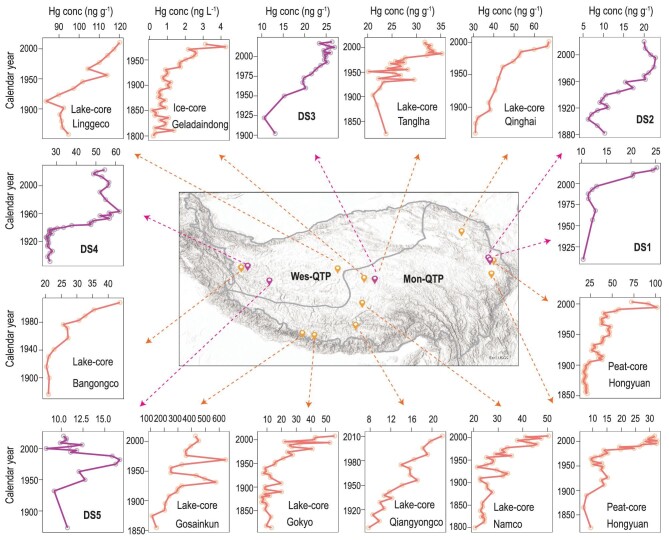
Variation in the Hg concentrations in selected cores (DS1−DS5) and reported Hg concentrations in lake sediment, ice and peat cores in earlier studies [16,18,30–33].

The vertical Hg trends of DS1, DS2 and DS3 are similar to trends in earlier archives of the Mon-QTP. However, the trends of DS4 and DS5 are different from earlier reports (Fig. 2). The Hg vertical trends in the archives of the Mon-QTP confirm a distinct increase in anthropogenic Hg accumulation during last 100−150 years. Additionally, the Wes-QTP is more sensitive to global warming than the Mon-QTP due to its elevation, lower precipitation and temperature [35]. Wetlands are transition zones where the flow of water and the changes in hydrology and vegetation would distinctly influence Hg accumulation in sediments. Therefore, most sites in the Wes-QTP show a subsurface Hg peak and only one site, as shown in Fig. 1b, shows a decreasing downwards trend. This suggests additional environmental disturbances at most sites of the Wes-QTP, as discussed below.

### Isotopic evidence for Hg sources

There are several sources for Hg accumulation in sediments of wetlands. One is the vegetation uptake of atmospheric Hg^0^, which exhibits negative Δ^199^Hg (–0.35‰ to –0.15‰) and slightly negative Δ^200^Hg (–0.10‰ to 0.00‰) signatures [28,36,37]. Another is atmospheric Hg^2+^ inputs, which show highly positive Δ^199^Hg values (≤0.6‰−1.0‰) and positive Δ^200^Hg values (0.15‰−0.30‰) in the QTP [38,39]. The geogenic Hg from rock weathering also likely contributes to Hg sources in sediments and this endmember shows values of Δ^199^Hg and Δ^200^Hg that are close to zero [20]. Soil erosion is a mixed Hg source. It contains Hg from previously deposited atmospheric Hg^2+^, primary Hg^0^ in glacier and permafrost soil, and geogenic Hg, which exhibits with a wide range of Hg isotopic signatures [21].

**Figure 3.**
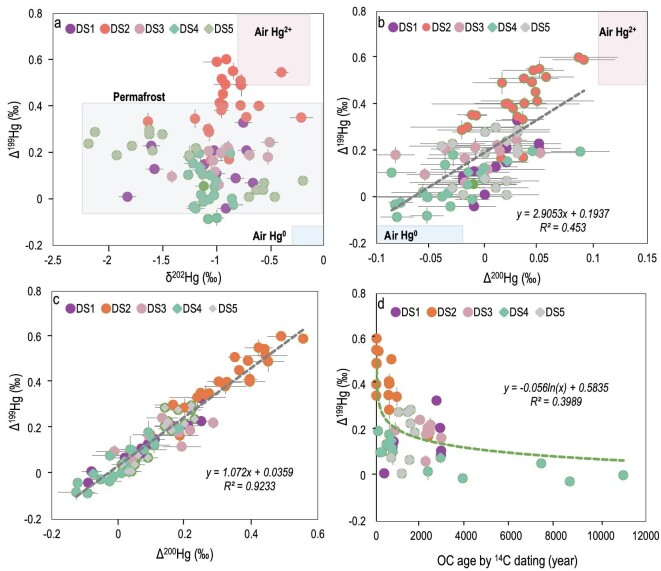
Hg isotopic composition in the selected sediment cores. (a) Δ^199^Hg versus δ^202^Hg, (b) Δ^199^Hg versus Δ^200^Hg, (c) Δ^199^Hg versus Δ^201^Hg and (d) Δ^199^Hg versus OC (organic carbon) age. The error bar in (a−d) represents 1 standard error. The Hg isotopic signatures in (a) and (b) for air Hg^0^ and air Hg^2+^ and the permafrost soils are derived from our earlier study [21].

**Figure 4.**
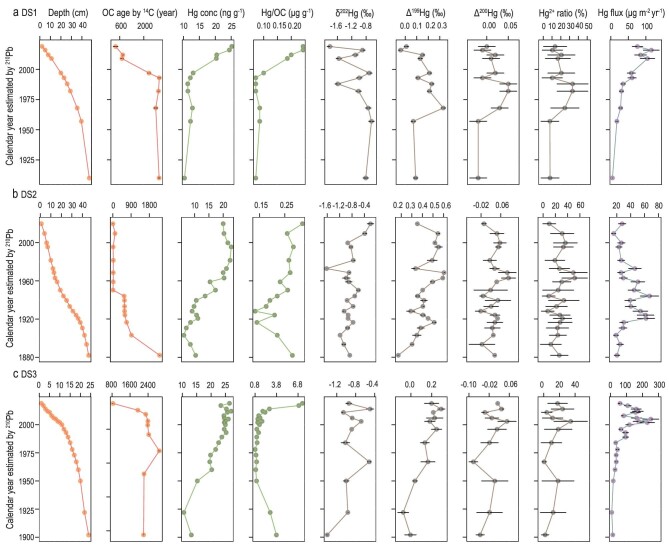
The ^210^Pb- and ^14^C-dated chronology, Hg concentration, Hg/OC ratio, isotopic compositions (δ^202^Hg, Δ^199^Hg and Δ^200^Hg), atmospheric Hg^2+^ contribution and Hg flux in (a) DS1, (b) DS2 and (c) DS3 cores. The error bar represents 1 standard error. The detailed data can be found in Table S1.

**Figure 5.**
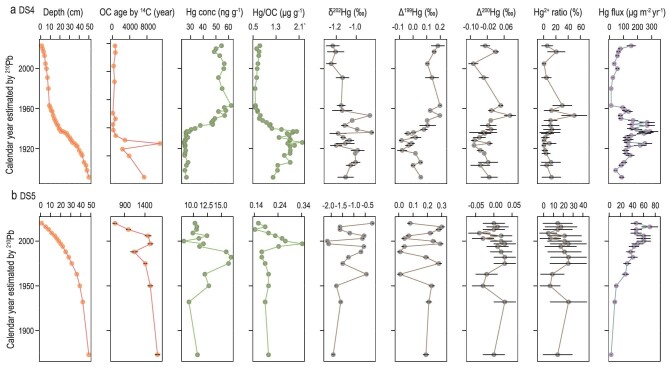
The ^210^Pb- and ^14^C-dated chronology, Hg concentration, Hg/OC ratio, isotopic compositions (δ^202^Hg, Δ^199^Hg and Δ^200^Hg), atmospheric Hg^2+^ contribution and Hg flux in (a) DS4 and (b) DS5 cores. The error bar represents 1 standard error. The detailed data can be found in Table S1.

We observed distinct positive Δ^199^Hg values in sediments (Fig. 3a). Biogeochemical processes such as MeHg photodegradation cause odd-MIF [40]. Given the absence of sunlight and the low MeHg/THg ratio (<10%) in sediments [41], it is unlikely that *in situ* MeHg photodegradation or Hg^2+^ photoreduction can lead to a large shift in Δ^199^Hg. This is further confirmed by the slope of Δ^199^Hg versus Δ^201^Hg, which has a value of 1.07 (Fig. 3c) and is largely different from the slope of 1.5−1.6 induced by MeHg photodegradation [40]. Δ^200^Hg is a superior tracer for atmospheric Hg^2+^ input [42–44]. The strong correlation between Δ^200^Hg and Δ^199^Hg (Fig. 3b) confirms the importance of atmospheric Hg^2+^ accumulation. The dated cores in the Mon-QTP have significantly higher Δ^200^Hg (0.02 ± 0.03‰ versus –0.01 ± 0.03‰) and Δ^199^Hg (0.28 ± 0.17‰ versus 0.10 ± 0.11‰) values than cores in the Wes-QTP (*P* < 0.05). This is consistent with the higher precipitation with atmospheric Hg^2+^ input in the Mon-QTP (21.4 ± 11.7% versus 13.0 ± 13.0% in Figs 4 and 5; *P* < 0.05). The Δ^199^Hg value increases with the decreasing age of organic carbon in the sediment (Fig. 3d). This is because of the younger age of the organic carbon in the Mon-QTP influenced by atmospheric Hg^2+^.

Soil erosion from the upstream terrestrial ecosystem could contribute to the Hg accumulation in sediments. The old age of organic carbon compared with the sediment age suggests that organic soil erosion is the main source of wetland sediment. The ^210^Pb dating of DS1, DS3, DS4 and DS5 shows that the sediment age covers the most recent 110−150 years, while ^14^C dating shows that the organic carbon age ranges from 360 to 2900 years (Figs 4 and 5). DS2 shows modern organic carbon at the 20-cm core depth (i.e. formation after the year of 1950) and organic carbon that is aged >500 years below the 20-cm depth (Fig. 4b). Thus, the legacy Hg tends to be absorbed by organic compounds, then transported and accumulated in sediments via soil erosion. The similar Hg isotopic signatures between sediments and upstream permafrost (Fig. 3a) confirm this hypothesis. The soil erosion from the thawing permafrost and hydrology-induced sedimentation changes also contribute to the observed variations in δ^202^Hg (Fig. 3a). The source contribution of Hg in the uppermost soil has been documented as being 40%−55% from atmospheric Hg^0^ deposition, 23%−25% from precipitation Hg^2+^ and 23%−38% from geogenic sources [21]. Hence, soil erosion mobilizes the atmospheric Hg^2+^ and other Hg sources, and facilitates Hg accumulation in the sediments (Figs 4 and 5). Vegetation uptake of atmospheric Hg^0^ and geogenic Hg are additional sources. Specifically, the change in vegetation cover in the wetlands has contributed to the fluctuation of δ^202^Hg (–2.2‰ to –0.2‰; Figs 4 and 5), as vegetation uptake of atmospheric Hg^0^ is known to produce a large MDF (–2.6‰ to –2.8‰ for ^202^Hg) [28,36,37].

### Processes responsible for a ‘surface peak’ in Hg accumulation

We depict the Hg accumulation processes and sources in Core DS1, DS2 and DS3 in more detail to understand the mechanisms resulting in a surface peak pattern in the Mon-QTP. Cores DS1 (102.421°E, 34.205°N and 3476 m of elevation) and DS2 (102.554°E, 33.985°N and 3432 m of elevation) were taken in Ruoergai Wetland, the largest wetland area in the QTP. Core DS3 represents a proglacial wetland in Mt. Tanglha (91.972°E, 32.925°N) with an elevation of 5140 m.

Figure 4a−c shows that sediments before the 1950s consisted of organic carbon that was ≤500−2900 years old at DS1, DS2 and DS3, and had a low Hg concentration, Hg/organic carbon (OC) ratio (the ratio of Hg over organic carbon) and variations in δ^202^Hg, Δ^199^Hg and Δ^200^Hg signatures. These suggest a steady sedimentation process before the 1950s. The old age of organic matter has a consistent Hg/OC ratio due to the long-term complexation of Hg and organic matter. The Hg concentration in the three cores has increased by 100%−150% since the 1950s. The Hg/OC ratio has also doubled. The δ^202^Hg, Δ^199^Hg and Δ^200^Hg values fluctuated greatly over the same period. The Hg levels and isotopic compositions in the three cores have synchronously varied in the past 50−70 years, suggesting perturbations of Hg biogeochemical processes.

By using the Δ^200^Hg value as the tracing signal for the precipitation Hg^2+^ input, it is found that a peak in atmospheric Hg^2+^ contribution (35%−55%) at the DS1 and DS2 sites occurred during the 1960s to 1980s (Fig. 4a and b). The atmospheric Hg^2+^ input includes precipitation Hg and legacy Hg from soil erosion. Soil erosion contributed to the peak in atmospheric Hg^2+^ accumulation in the 1960s to 1980s at DS1 (Fig. 4a). The elevated wet deposition was likely the cause of this peak at DS2 due to the accumulation of modern organic matter (Fig. 4b). The decreasing trend of Δ^199^Hg in DS1 and DS2 since the 2000s can be attributed to changes in hydrological conditions and anthropogenic impacts. Specifically, the increasing anthropogenic Hg emissions through long-term deposition with zero Δ^199^Hg values diluted (i.e. decrease) the Δ^199^Hg values in sediments [45]. This is consistent with the increasing Hg concentrations at DS1 and DS2 during this period.

Given that DS3 is a proglacial wetland and impacts from climate-induced hydrological fluctuation are more pronounced, as shown in Fig. 4c, the trends of the Hg concentration, Hg/OC ratio, Δ^199^Hg, Δ^200^Hg and atmospheric Hg^2+^ contribution have increased since the 1950s. There is a strong correlation between Hg concentration and Δ^199^Hg (*R*^2^ = 0.89, Fig. S5). Organic matter aged ≤840−2200 years has accumulated in the sediment (Fig. 4c). This is driven by global warming that accelerates the melting of glaciers and permafrost [30,35], which releases the legacy Hg sequestered in the ice and permafrost soil into wetlands. Additionally, the increasing atmospheric Hg^2+^ deposition caused by anthropogenic emissions and climate-induced increase in vegetative biomass also contribute to the increasing trend of Hg [11].

### Processes responsible for a ‘subsurface peak’ in Hg accumulation

DS4 is a marsh site (80.387°E, 33.707°N and 4438 m of elevation) and DS5 is a lacustrine site (82.476°E, 32.561°N and 4363 m of elevation). The Hg concentrations at DS4 and DS5 have not shown increasing trends since the Industrial Revolution, as recorded in the lake sediment cores of the Wes-QTP (Figs 2 and 5). The Hg concentration at DS4 increased from 30−40 to 50−62 ng g^−1^ during the period of the 1940s to the 1960s and has been nearly constant, with values of 50−60 ng g^−1^, since then (Fig. 5a). The Hg concentration at DS5 increased from the 1960s and peaked in the 1980s, and the Hg/OC ratio peaked in the 2000s (Fig. 5b).

In addition to the impact of anthropogenic Hg deposition, the regional environmental changes in the last 50−70 years have likely contributed to changes in Hg accumulation in the wetlands in QTP. In the Wes-QTP, ice melting in the cryosphere is the primary water source for wetlands [35]. The subsurface peak in the Hg concentration is likely due to the hydrological changes that cause soil erosion and changes in the sedimentation rate. The Δ^199^Hg and Δ^200^Hg peaks are associated with highly variable Hg concentrations and Hg/OC ratios found at SD4 and SD5 (Fig. 5a and b). The isotopic signals are associated with elevated atmospheric Hg^2+^ contributions (30%−50% at DS4 and 25%−34% at DS5). These suggest non-steady sedimentation conditions in the 1940s to 1980s. Finally, this period is consistent with the time at which global warming forced changes in the hydrological processes in the Wes-QTP [13,35]. There is a significant anti-correlation (*P* < 0.01, Fig. S6) between δ^202^Hg with Δ^199^Hg at SD4 and SD5. This cannot be explained by atmospheric Hg deposition changes that induce a positive correlation [28,46]. Given the –1.0‰ to 0 value of δ^202^Hg and 0‰–0.40‰ value of Δ^199^Hg in the upstream permafrost [21], soil erosion caused by the thawing permafrost is the most likely cause of the negative correlation of δ^202^Hg with Δ^199^Hg.

### Increasing Hg accumulation flux

The Hg accumulation flux at DS1 changed dramatically from ∼5 μg m^−2^ yr^−1^ in the 1910s to 28−31 μg m^−2^ yr^−1^ in the 1960s to 1980s and to 104−115 μg m^−2^ yr^−1^ in the 2010s (Fig. 4a). Likewise, the Hg accumulation flux at DS3 increased from 9−18 μg m^−2^ yr^−1^ during the 1900s to 1950s to 118−252 μg m^−2^ yr^−1^ in the 2000s (Fig. 4c). The flux of ≤10−20 times since the 1910s at DS1 and DS3 cannot be explained by the increase in the Hg concentration (only ∼2.5 times). The magnitude of the flux increase is also much higher than those found in QTP ice cores, which show an increase of two to seven times over the same period [16–18]. Given the organic matter (>500 years) accumulated in sediments at DS1 and DS3 (Fig. 4a and b), we conclude that melting glaciers and thawing permafrost due to ongoing climate warming increased water and sediment discharges in the region [47,48], and greatly enhanced Hg accumulation in the Mon-QTP wetland sediments. The peak in Hg accumulation flux occurred during the 1920s to 1960s at DS2 (40−67 μg m^−2^ yr^−1^) due to the elevated sediment accumulation rate (Fig. 4b).

Although the Hg concentration has shown a small increase in the last 40−50 years (Fig. 5a and b), the increased sedimentation rate since the 1960s at DS4 enhanced the Hg accumulation flux by ≤10 times (from 12 to 154 μg m^−2^ yr^−1^) in the 2010s and by ≤15 times (from 5 to 76 μg m^−2^ yr^−1^) at DS5. Ice melting and permafrost thawing caused by global warming have increased water and sediment discharges in the Wes-QTP [13,35]. Additionally, the local hydrological changes increased sediment accumulation rates at DS4 during the 1920s to 1960s, resulting in Hg accumulation peaks (240−327 μg m^−2^ yr^−1^).

### Environmental implications

This study provides insight into understanding the Hg cycle in the QTP. First, the combined effects of human impacts and global warming in monsoon-controlled regions have accelerated Hg accumulation in wetlands, posing a significant risk to the local ecosystems. Such ecological risk is distinctly pronounced in proglacial wetlands, as melting glaciers and permafrost release sequestered Hg into the wetlands that subsequently accumulates in the sediments. The harsh climate in westerly-controlled regions leads to a fragile wetland ecosystem. Changes in climate and hydrological processes in these regions have influenced the Hg accumulation in wetlands. The amplified legacy Hg input through soil erosion has also contributed to elevated Hg accumulation. As anthropogenic Hg emissions are being regulated through the Minamata Convention of Mercury, the Hg concentration in the atmosphere is expected to decrease continuously in the foreseeable future, as has been observed at remote sites in the northern hemisphere [49,50]. The lowered concentration could not directly reduce Hg accumulation flux in wetlands of the QTP due to the legacy Hg input from melting glaciers and thawing permafrost. This work shows that glacier melting and permafrost thawing forced by global warming, coupled with human influences, alter geomorphology and hydrology, affect Hg transport and accumulation, and pose ecological risks in wetlands in the QTP.

## METHODS

### Sample collections and measurements

Detailed information regarding the study sites, sampling, dating and analytical methods is provided in Text S1–S3. The activities of ^210^Pb, ^226^Ra and ^137^Cs were measured by using gamma spectrometry (Canberra GX6020). Ages and sedimentation rates were estimated by using the constant flux dating model (also called Constant Rate of Supply). Then, we used the ^137^Cs activity profiles to validate ^210^Pb chronologies based on the ^137^Cs distribution pattern along a sediment core, in which the maximum value is assumed to be related to 1963. Radiocarbon measurements were performed on a 1-MV accelerator mass spectrometry (i.e. AMS 3SDH-1) at the State Key Laboratory of Environmental Geochemistry, Institute of Geochemistry, Chinese Academy of Sciences. The Hg isotopic ratios for all samples were determined by using a Nu-Plasma II multicollector-inductively coupled plasma mass spectrometer. The Hg-MDF is reported in δ notation referenced to the neighboring NIST-3133 solution:


(1) \begin{eqnarray*}
&&\!\!{{\delta }^{202}}{\mathrm{Hg}}\left( {}^{0}\!/\!_{00} \right)= 1000 \\
&& \times \left[ {\left( {^{202}{\mathrm{Hg}}{{/}^{198}}{\mathrm{H}}{{{\mathrm{g}}}_{{\mathrm{sample}}}}} \right)/\left( {^{202}{\mathrm{Hg}}{{/}^{198}}{\mathrm{H}}{{{\mathrm{g}}}_{{\mathrm{NIST}} - 3133}}} \right) - 1} \right].\\
\end{eqnarray*}

The MIF is reported as Δ^xxx^Hg:


(2) \begin{eqnarray*}
{{\Delta }^{199}}{\mathrm{Hg}}\left( {}^{0}\!/\!_{00} \right) = {{\delta }^{199}}{\mathrm{Hg}} - 0.2520 \times {{\delta }^{202}}{\mathrm{Hg}}.
\end{eqnarray*}

(3) \begin{eqnarray*}
{{\Delta }^{200}}{\mathrm{Hg}}\left( {}^{0}\!/\!_{00} \right) = {{\delta }^{200}}{\mathrm{Hg}} - 0.5024 \times {{\delta }^{202}}{\mathrm{Hg}}.
\end{eqnarray*}

(4) \begin{eqnarray*}
{{\Delta }^{201}}{\mathrm{Hg}}\left( {}^{0}\!/\!_{00} \right) = {{\delta }^{201}}{\mathrm{Hg}} - 0.7520 \times {{\delta }^{202}}{\mathrm{Hg}}.
\end{eqnarray*}

### Hg isotope mixing model

Three source endmembers for the Hg accumulation in the sediments are parameterized: atmospheric Hg^0^ deposition, atmospheric Hg^2+^ and geogenic Hg weathering from rocks. The MeHg photodegradation or Hg^2+^ photoreduction may lead to odd-MIF. Hence, the Δ^199^Hg signatures cannot be directly used to quantify the contribution of the atmospheric Hg sources. Since known Hg biogeochemical processes in wetlands do not lead to even-MIF [28,37], Δ^200^Hg is a superior tracer to identify the atmospheric Hg^2+^ deposition sources. Therefore, Δ^200^Hg signals were utilized for estimating the contribution of rainfall inputs (i.e. atmospheric Hg^2+^) and non-rainfall inputs, as follows:


(5) \begin{eqnarray*}
&&{{\Delta }^{200}}{\mathrm{H}}{{{\mathrm{g}}}_{\textit{rainfall}}} \times {{f}_{\textit{rainfall}}} + {{\Delta }^{200}}{\mathrm{H}}{{{\mathrm{g}}}_{non - \textit{rainfall}}} \\
&&\quad \times ( {1 - {{f}_{\textit{rainfall}}}} ) = {{\Delta }^{200}}{\mathrm{H}}{{{\mathrm{g}}}_{\textit{soil}}},
\end{eqnarray*}

where Δ^200^Hg*_rainfall_* is the signature of the atmospheric Hg^2+^ inputs and obtained from observations in the QTP [38,39] and *f_rainfall_* is the atmospheric Hg^2+^ input contribution. The Δ^200^Hg signatures of air Hg^2+^ and air Hg^0^ in the QTP were 0.19 ± 0.05‰ and −0.06 ± 0.03‰, respectively [20]. We used the Monte Carlo simulation to quantify uncertainties of the Hg isotopic mixing model. These uncertainties are quantified by generating 1 million groups of Δ^200^Hg signatures randomly ranging from mean – SD to mean + SD to solve Equation (5).

### Statistical analysis

Data were analysed by using the statistical programs R 4.10 and ArcGIS Pro. We used the paired samples *t*-test, one-Way Analysis of Variance and post hoc Tukey’s Honestly Significant Difference test to conduct the significant difference analysis when data were normally distributed. Otherwise, the Kruskal–Wallis test was applied. Additionally, we used the polynomial curve fitting (order = 6) to simulate the trend of the vertical Hg concentration in cores. The confidence level for all statistics is 95%.

## Supplementary Material

nwae414_Supplemental_File

## Data Availability

The Supplementary Data can be found in TPDC repository (https://data.tpdc.ac.cn/en/data/70b7839b-76d7-4261-8923-6f7b40ed5970).
