# Supplementary material for: Influence of global warming and human activity on mercury accumulation patterns in wetlands across the Qinghai–Tibet Plateau
Source: Natl Sci Rev. 2024 Nov 15;12(1):nwae414. doi: 10.1093/nsr/nwae414 (PMC11707873; doi:10.1093/nsr/nwae414)
Supplement: nwae414_Supplemental_File [file nwae414_supplemental_file.docx]

Supplementary Data to:

**Influence of global warming and human activity on mercury accumulation patterns in wetlands across the Qinghai-Tibet Plateau**

Xinbin Feng ^1,2#*^, Xun Wang ^1#^, Longyu Jia ^1,2^, Wei Yuan ^1^, Meng Lu ^3^, Nantao Liu ^1^, Fei Wu ^1,2^, Xinyuan Cai ^1,2^, Feiyue Wang ^4^, Che-Jen Lin ^5^

^1^ State Key Laboratory of Environmental Geochemistry, Institute of Geochemistry, Chinese Academy of Sciences, Guiyang 550081, China.

^2^ University of Chinese Academy of Sciences, Beijing 100049, China

^3^ School of Ecology and Environmental Science, Yunnan University, Kunming 650091, China

^4^ Centre for Earth Observation Science, and Department of Environment and Geography, University of Manitoba, Winnipeg, MB R3T 2N2, Canada

^5^ Center for Advances in Water and Air Quality, Lamar University, Beaumont, TX, 77710, United States

^#^ These authors contributed equally

* Corresponding Author:

Xinbin Feng; Email: fengxinbin@vip.skleg.cn

**This file includes:**

Text S1 to S3

Figures S1 to S6

Table S1

**Supplementary Data Text**

**Text S1: Sample collection**

This study mainly focused on lacustrine marshes and wetlands and riverine wetlands of the QTP. The distribution of lacustrine marshes and wetlands for the year 2020 was sourced from the Third Pole Environment Data Center (<https://doi.org/10.11888/Terre.tpdc.272305>, with total surface area of 7280 km^2^). The data on riverine wetlands was acquired from the Sustainable Wetlands Adaptation and Mitigation Program (SWAMP, <https://doi.org/10.17528/CIFOR/DATA.00058>, total area of 158 km^2^). Annual precipitation in the region ranges from 50 mm to 2500 mm, decreasing gradually from the southeast to the northwest (Figure 1A).

We collected 49 sediment cores from June to October in 2020 including 14 riverine wetland sites, 21 lake sites, and 14 marsh sites (Figure S1). The site selection is based on the river, lake and marsh distribution, and their sizes and traffic accessibility. The sampling locations are in the middle of land and water transitional zones, with typical wetland vegetation species such as *Blysmus sinocompressus* and *Kobresia tibetica*. The elevation of the sampling sites ranges 2631 m to 5143 m above the sea level, and 63% of the sites are above 4000 m. Each wetland sediment core was drilled to a depth of 50 cm with a peat auger. All core samples were sectioned at a 1-cm interval and then placed in a clean polyethylene bag and immediately stored in a 4°C portable refrigerator.

**Text S2: Sediments and ice core chronology**

**^210^Pb dating.** The DS1 to DS5 cores samples were freeze-dried and passed through a 2 mm sieve. They were then sealed hermetically into cylindrical containers for more than two weeks to achieve equilibrium between ^226^Ra and its decay products. The activities of ^210^Pb, ^226^Ra and ^137^Cs were measured by gamma spectrometry (Canberra GX6020) at the State Key Laboratory of Environmental Geochemistry, Institute of Geochemistry, Chinese Academy of Sciences. We then mixed three 1-cm interval samples into one sample to obtain a mixed sample of at least 10 g. The counting time was 24 h per sample, providing a precision of approximately ±10% at the 90% confidence level for the gamma ray measurements. The excess ^210^Pb (^210^Pb_ex_) was estimated from activities of ^210^Pb and ^226^Ra:

^210^Pb_ex_ = ^210^Pb - ^226^Ra (1)

Ages and sedimentation rates were estimated by using the constant flux dating model (CF also called Constant Rate of Supply, CRS). The main assumption of the CF model is that the ^210^Pb_ex_ flux to the water-sediment interface remains constant, while the sedimentation rate may vary in time (1). The sedimentary age (t_i_) was estimated by:

$t_{i}=\frac{1}{\lambda}ln(\frac{I_{i}}{I})$ (2)

where $\lambda$ is the ^210^Pb day constant, and I is the total ^210^Pb_ex_ inventory in the top 50-cm depth, and I_i_ is the ^210^Pb_ex_ inventory below the layer being dated. Finally, we used the ^137^Cs activity profiles to validate ^210^Pb chronologies based on the ^137^Cs distribution pattern along a sediment core, in which the maximum value is assumed to be related to 1963–1964, the period of maximum atmospheric fallout ^137^Cs.

**^14^C dating.** Radiocarbon measurements were performed at the State Key Laboratory of Environmental Geochemistry, Institute of Geochemistry, Chinese Academy of Sciences. The samples were synthesized graphite using a Carbon Extraction & Graphitization Systems (Aeon CEGS 12X). The synthesized graphite was transferred and pressed into a special aluminum holder, which was used for the measurement of ^14^C on a 1 MV accelerator mass spectrometry (i.e., AMS 3SDH-1). Radiocarbon values are reported as Δ^14^C, the part per thousand deviation of the sample's ^14^C/^12^C ratio relative to a nineteenth century wood standard. Δ^14^C was corrected for fractionation using δ^13^C of samples according to Polach and Stuiver (2). Δ^14^C is expressed as:

Δ^14^C = [R_sample_/R_standard_ −1] × 1000 (3)

where R is ^14^C/^12^C. NBS Oxalic acid II (SRM-4990C) and IAEA-C7 were used as reference standards. Instrumental precision of Δ^14^C analysis was based on the multiple analyses on a target, with a SD ranging normally from 2‰ to 5‰. Finally, we used calibration programs OxCal to determine the calendar year.

**Text S3: Chemical analysis**

Samples were transported to the laboratory and then freeze-dried to a constant weight. All dried samples were grinded by an agate grinder and sieved with a 200‐mesh sieve. Mercury concentrations in sediment were measured by a DMA80 Hg analyzer (3, 4). The certified reference material (CRM) of GBW07405 (GSS-5, soil, Hg = 290 ± 40 ng g^-1^) from the National Standard Reference Materials of China was used for QA/QC (Quality assurance / Quality control) and measured every nine samples with a recovery of 95-105%. An Elementar Vario Macro Cube analyzer (Germany, limit of detection ≤10 ppm) was used to measure total carbon (C) and nitrogen (N). The B2150 and AR2026 was used as CRMs for C and N, with for an average precision of 0.2%.

Before the Hg isotopic measurement, all samples were processed by a double-stage tube furnace and trapping solutions (anti aqua regia, HNO_3_:HCl = 2:1, v/v) for Hg preconcentration. The QA/QC for the Hg isotopic measurement has been documented in our earlier studies (3-5). Briefly, the Hg concentration enriched in the trapping solution was measured by cold vapor atomic fluorescence spectrometry (Tekran 2500) following the US-EPA method 1631, with the pre-concentration recovery of 91~104%. The resulted Hg solutions were finally diluted to 0.5 ng mL^-1^ prior to Hg isotope measurement by the Nu-Plasma II MC-ICP-MS. The Hg-MDF is reported in δ notation referenced to the neighboring NIST-3133 solution:

δ^202^Hg (‰) =1000 × [(^202^Hg/^198^Hg_sample_)/(^202^Hg/^198^Hg_NIST-3133_) - 1] (4)

The MIF is reported as Δ^xxx^Hg following Blum and Bergquist (6):

Δ^199^Hg (‰) = δ^199^Hg − 0.2520 × δ^202^Hg (5)

Δ^200^Hg (‰) = δ^200^Hg − 0.5024 × δ^202^Hg (6)

Δ^201^Hg (‰) = δ^201^Hg − 0.7520 × δ^202^Hg (7)

The CRM of GSS-4 (soil) were combusted in the oven-enrichment system at a frequency of every 10 samples to test the potential isotopic bias. The recoveries of Hg preconcentrating were in the range of 92-104% for the CRM and the sediment samples. The Hg isotopic signatures for GSS-4 as δ^202^Hg=-1.72±0.16‰, Δ^199^Hg =-0.34±0.06‰, Δ^200^Hg=-0.00±0.04‰ and Δ^201^Hg =-0.34±0.06‰ (Mean±2SD, Standard deviation, n=6) (n=6). The NIST-8610 was also analyzed every 10 samples throughout the Hg isotope measurement, with the isotopic signatures of δ^202^Hg=-0.53±0.08‰, Δ^199^Hg=-0.00±0.08‰ and Δ^200^Hg=-0.03±0.06‰ (n=12). All the measured Hg isotopic signatures of the CRM were consistent with standard values (6, 7).

**Supplementary Data Figures**


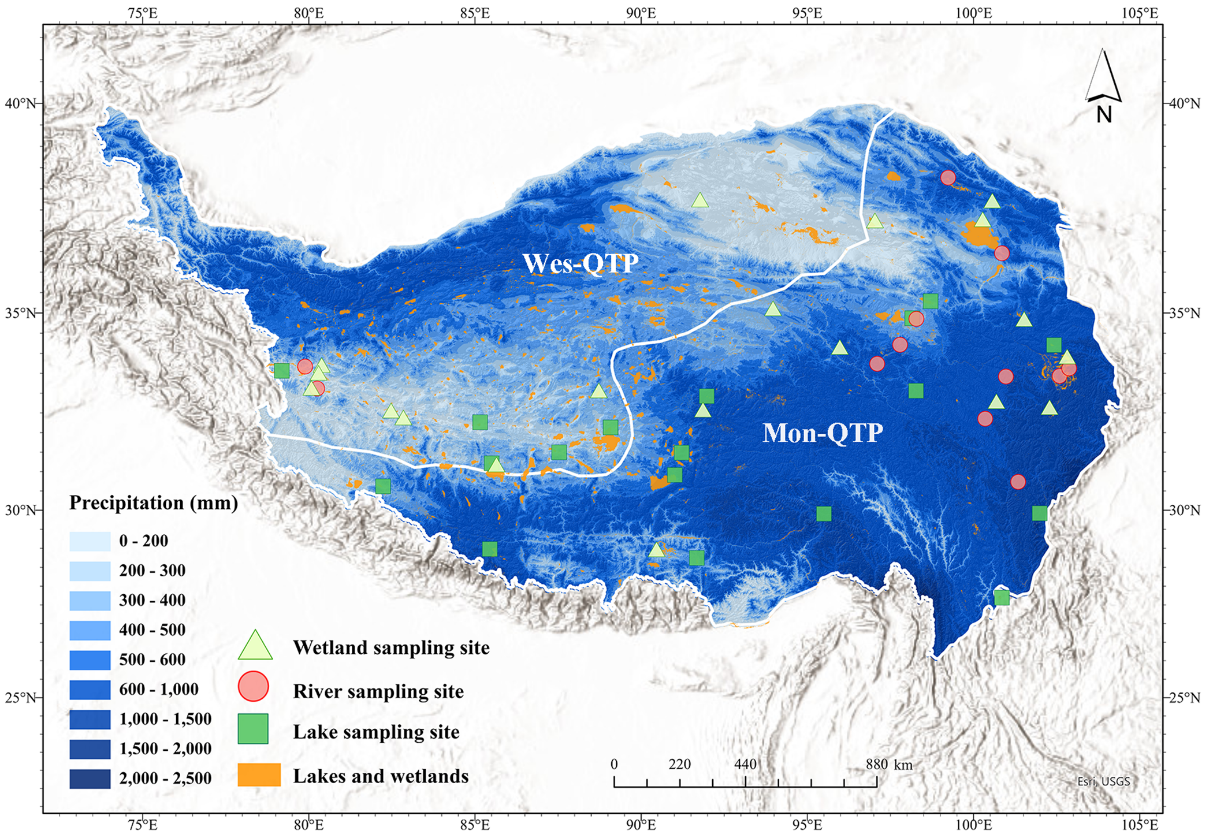


Fig. S1. Sampling sites on the Qinghai-Tibet Plateau.


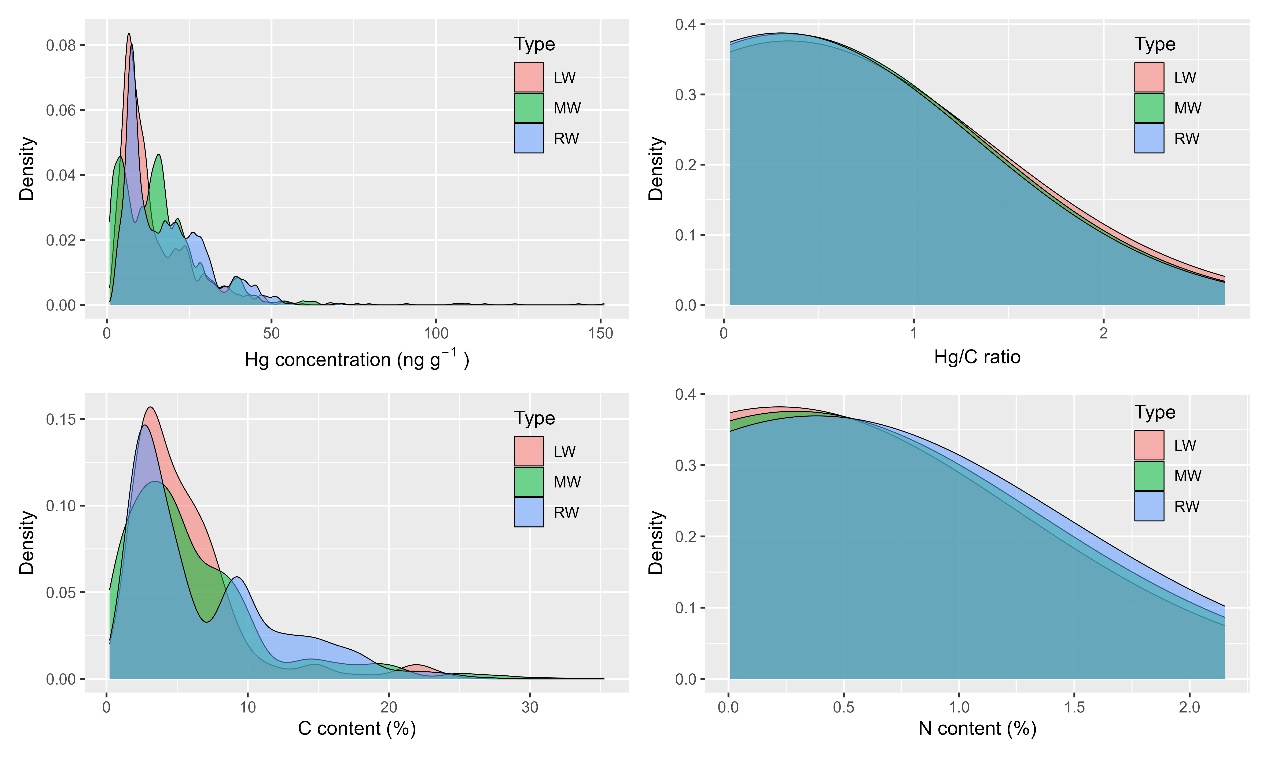


Fig. S2. The density plots of Hg concentration, Hg/C ratio (μg g^-1^), C content and N content among LW (lake wetland), RW (Riverine wetland) and MW (marsh).


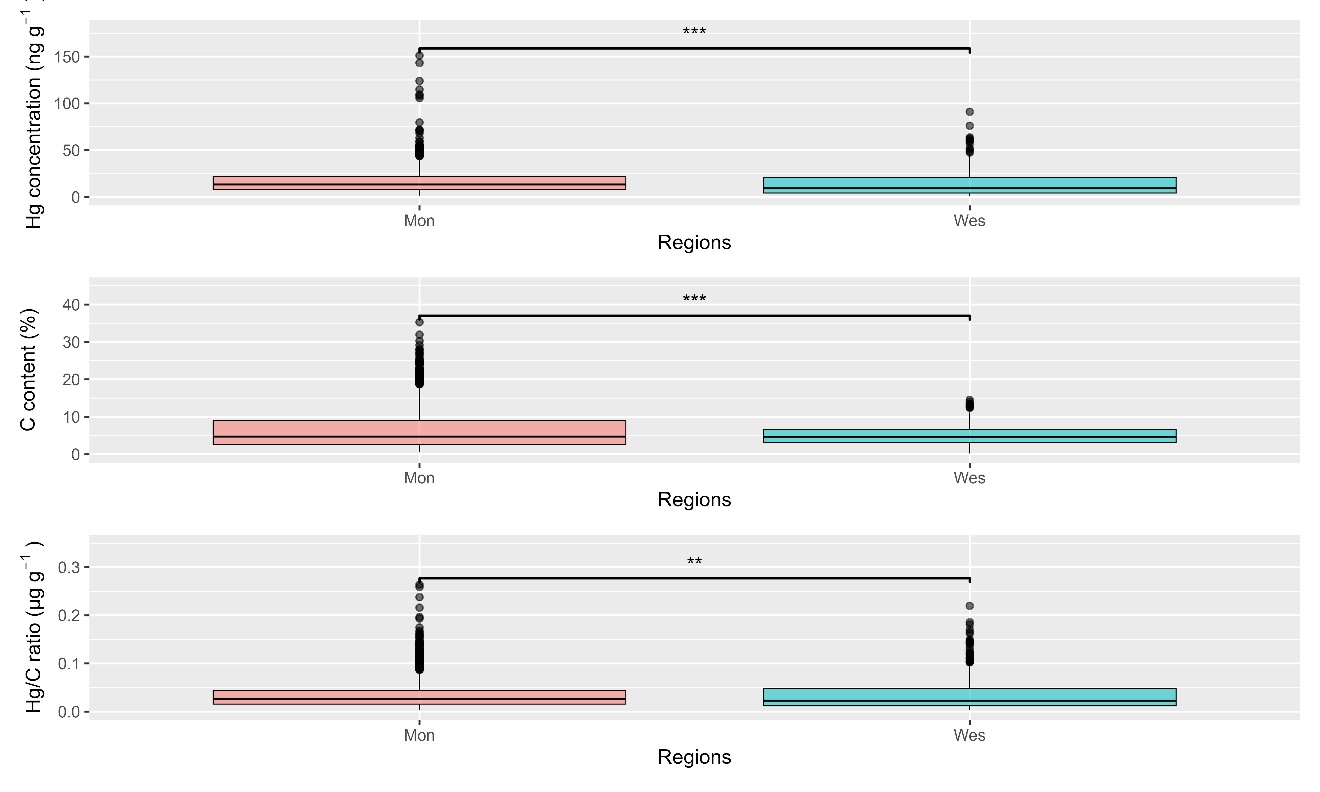


Fig. S3. The box chart plots of the concentrations of Hg, C and ratio of Hg/C between monsoon-controlled region (Mon) and westerly-controlled region (Wes) of the Qinghai-Tibet Plateau. “*” means p value <0.05, and “**” means p<0.01, and “***” means p < 0.001.


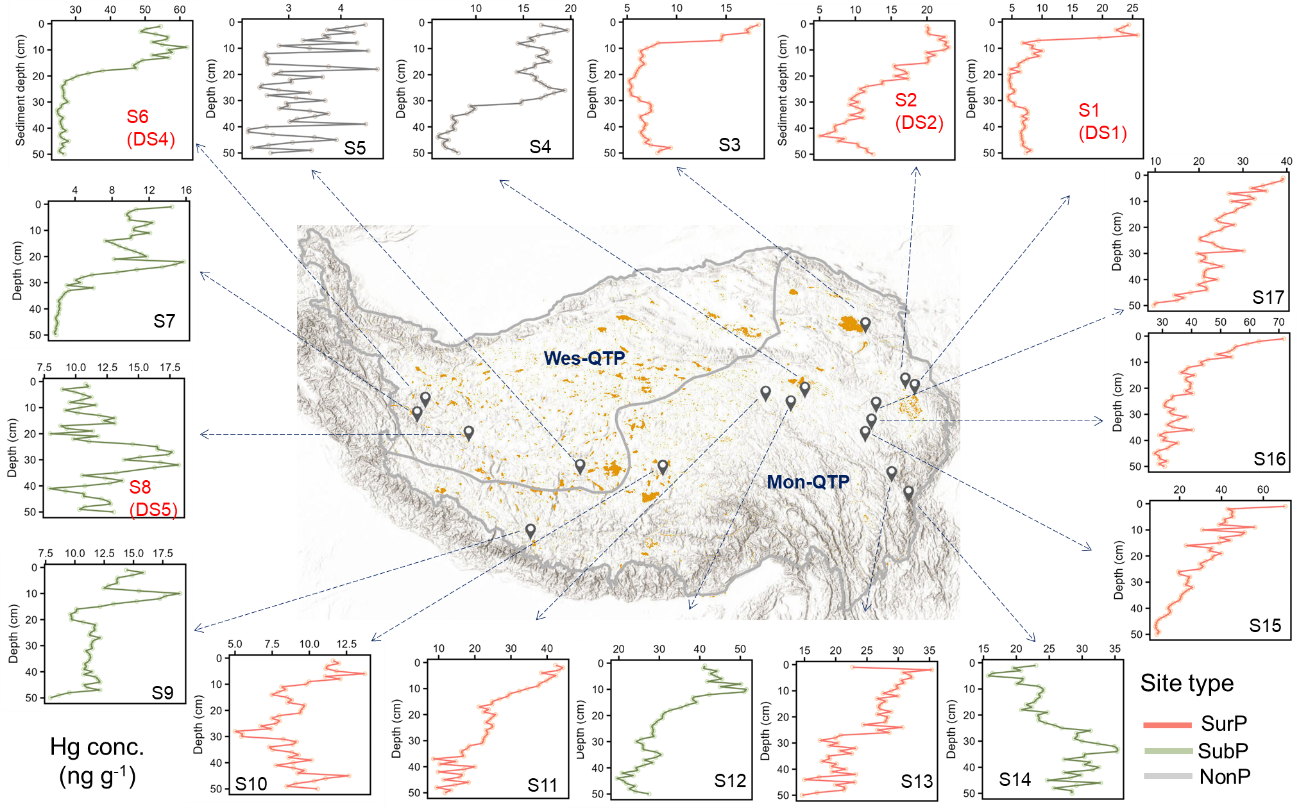


Fig. S4. Variation of the Hg concentration in selected cores with a total C content of > 5%. SurP, SubP and NonP are Surface Peak, Subsurface Peak, and Non-consistent Peak sites, respectively. DS1, DS2, DS4 and DS5 are mentioned as the dated cores in Figure 2-5.


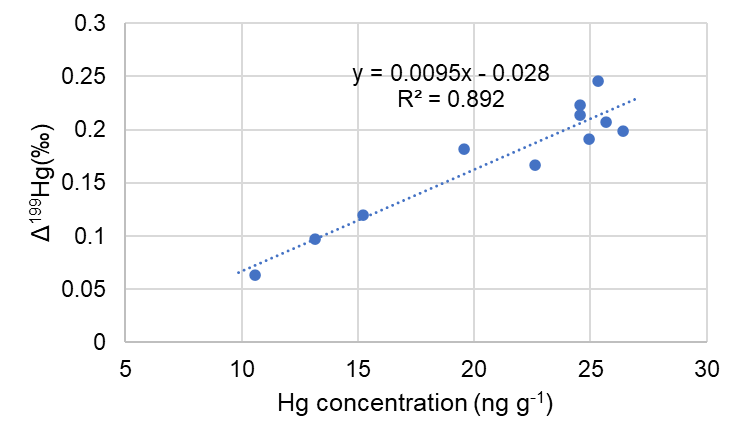


Fig. S5. The Hg concentration versus Δ^199^Hg in the cores of SD3.


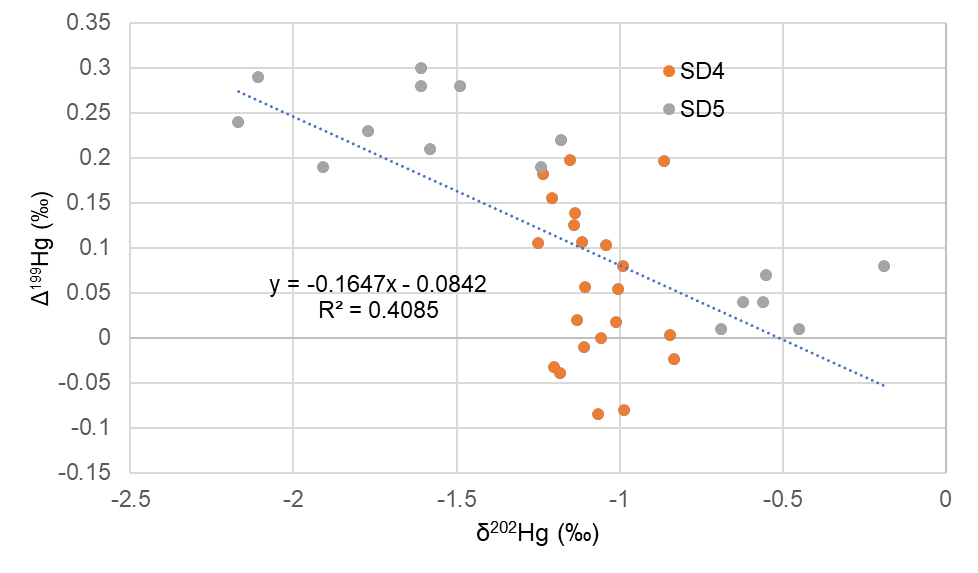


**Fig. S6.** The δ^202^Hg versus Δ^199^Hg in the cores of SD4 and SD5.

Table S1. The ^210^Pb- and ^14^C-dated chronology, Hg concentration, isotopic compositions (δ^202^Hg, Δ^199^Hg, and Δ^200^Hg), and Hg flux in DS1−DS5 cores

| Pb_Year | Depth | Site | Hg | δ^202^Hg | Δ^199^Hg | Δ^200^Hg | Δ^201^Hg | Hg_flux | F14C | C14_BP | C14_age | C14_SD |
| --- | --- | --- | --- | --- | --- | --- | --- | --- | --- | --- | --- | --- |
| 2019 | 2 | DS1 | 25 | -1.81 | 0.01 | 0.00 | -0.08 | 75.89 | 0.9699 | 291 | 362 | 138 |
| 2016 | 5 | DS1 | 24 | -0.90 | -0.04 | -0.01 | -0.09 | 115.44 |  |  |  |  |
| 2012 | 8 | DS1 | 20 | -1.10 | 0.15 | 0.02 | 0.11 | 84.67 | 0.9042 | 711 | 782 | 42 |
| 2009 | 11 | DS1 | 20 | -1.56 | 0.13 | 0.01 | 0.09 | 104.45 | 0.9152 | 662 | 733 | 79 |
| 1997 | 20 | DS1 | 13 | -0.71 | 0.21 | 0.02 | 0.16 | 58.36 | 0.7605 | 2231 | 2302 | 85 |
| 1993 | 23 | DS1 | 12 | -0.99 | 0.11 | -0.01 | 0.07 | 60.60 | 0.711 | 2825 | 2896 | 74 |
| 1988 | 26 | DS1 | 11 | -1.59 | 0.23 | 0.05 | 0.25 | 35.01 |  |  |  |  |
| 1982 | 29 | DS1 | 11 | -1.01 | 0.21 | 0.05 | 0.21 | 30.51 | 0.7163 | 2780 | 2851 | 47 |
| 1968 | 36 | DS1 | 13 | -0.74 | 0.33 | 0.03 | 0.25 | 27.87 | 0.7289 | 2621 | 2692 | 115 |
| 1957 | 40 | DS1 | 12 | -0.65 | 0.07 | -0.02 | 0.02 | 17.36 |  |  |  |  |
| 1910 | 47 | DS1 | 10 | -0.81 | 0.09 | -0.02 | 0.07 | 4.74 | 0.7122 | 2816 | 2887 | 47 |
| 2020 | 1 | DS2 | 20 | -0.21 | 0.35 | -0.01 | 0.27 | 27.67 | 1.0096 | Modern | 4 | 15 |
|  | 2 | DS2 | 20 |  |  |  |  | 28.65 |  |  |  |  |
|  | 3 | DS2 | 20 |  |  |  |  | 28.57 |  |  |  |  |
| 2010 | 4 | DS2 | 20 | -0.39 | 0.55 | 0.04 | 0.44 | 16.30 | 0.9958 | 35 | 107 | 15 |
|  | 5 | DS2 | 23 |  |  |  |  | 31.81 |  |  |  |  |
| 2001 | 6 | DS2 | 22 | -0.94 | 0.52 | 0.06 | 0.44 | 28.48 |  |  |  |  |
| 1997 | 7 | DS2 | 23 | -0.83 | 0.55 | 0.05 | 0.42 | 33.30 | 1.0355 | Modern | 12 | 20 |
|  | 8 | DS2 | 22 |  |  |  |  | 31.87 |  |  |  |  |
|  | 9 | DS2 | 23 |  |  |  |  | 44.25 |  |  |  |  |
| 1984 | 10 | DS2 | 22 | -0.76 | 0.49 | 0.02 | 0.45 | 29.65 | 1.0873 | Modern | 21 | 20 |
|  | 11 | DS2 | 20 |  |  |  |  | 43.78 |  |  |  |  |
| 1974 | 12 | DS2 | 21 | -1.62 | 0.33 | 0.03 | 0.24 | 31.32 |  |  |  |  |
| 1968 | 13 | DS2 | 20 | -0.90 | 0.60 | 0.09 | 0.49 | 37.09 | 1.0582 | Modern | 18 | 20 |
| 1963 | 14 | DS2 | 20 | -0.98 | 0.59 | 0.09 | 0.56 | 30.40 |  |  |  |  |
|  | 15 | DS2 | 20 |  |  |  |  | 53.84 |  |  |  |  |
| 1958 | 16 | DS2 | 15 | -0.91 | 0.50 | 0.04 | 0.39 | 43.01 | 1.056 | Modern | 17 | 15 |
|  | 17 | DS2 | 16 |  |  |  |  | 47.13 |  |  |  |  |
|  | 18 | DS2 | 17 |  |  |  |  | 35.43 |  |  |  |  |
| 1948 | 19 | DS2 | 17 | -0.60 | 0.40 | 0.02 | 0.33 | 43.94 | 1.0165 | Modern | 7 | 15 |
|  | 20 | DS2 | 15 |  |  |  |  | 40.99 |  |  |  |  |
|  | 21 | DS2 | 17 |  |  |  |  | 50.24 |  |  |  |  |
| 1940 | 22 | DS2 | 14 | -0.71 | 0.35 | -0.01 | 0.25 | 51.26 | 0.9516 | 484 | 556 | 15 |
|  | 23 | DS2 | 14 |  |  |  |  | 44.81 |  |  |  |  |
|  | 24 | DS2 | 12 |  |  |  |  | 53.39 |  |  |  |  |
| 1934 | 25 | DS2 | 11 | -0.96 | 0.41 | 0.05 | 0.40 | 24.36 | 0.9474 | 502 | 574 | 20 |
|  | 26 | DS2 | 11 |  |  |  |  | 32.38 |  |  |  |  |
|  | 27 | DS2 | 10 |  |  |  |  | 32.17 |  |  |  |  |
| 1925 | 28 | DS2 | 10 | -0.77 | 0.40 | 0.02 | 0.30 | 26.96 | 0.9462 | 506 | 578 | 20 |
|  | 29 | DS2 | 9 |  |  |  |  | 26.85 |  |  |  |  |
|  | 30 | DS2 | 10 |  |  |  |  | 33.45 |  |  |  |  |
| 1917 | 31 | DS2 | 9 | -1.06 | 0.29 | -0.02 | 0.20 | 23.56 | 0.9457 | 507 | 579 | 15 |
|  | 32 | DS2 | 10 |  |  |  |  | 53.70 |  |  |  |  |
|  | 33 | DS2 | 11 |  |  |  |  | 36.30 |  |  |  |  |
| 1910 | 34 | DS2 | 11 | -0.92 | 0.40 | 0.04 | 0.40 | 38.53 | 0.9409 | 521 | 593 | 15 |
|  | 35 | DS2 | 9 |  |  |  |  | 32.26 |  |  |  |  |
|  | 36 | DS2 | 11 | -0.93 | 0.45 | 0.05 | 0.36 | 31.86 |  |  |  |  |
|  | 37 | DS2 | 9 |  |  |  |  | 26.11 |  |  |  |  |
| 1894 | 38 | DS2 | 9 | -0.77 | 0.51 | 0.04 | 0.35 | 12.73 | 0.9305 | 606 | 678 | 15 |
|  | 39 | DS2 | 7 |  |  |  |  | 26.49 |  |  |  |  |
|  | 40 | DS2 | 7 | -0.94 | 0.38 | 0.03 | 0.32 | 10.06 |  |  |  |  |
|  | 41 | DS2 | 7 |  |  |  |  | 33.00 |  |  |  |  |
| 1843 | 42 | DS2 | 6 | -1.19 | 0.35 | 0.03 | 0.26 | 3.45 | 0.8881 | 846 | 918 | 15 |
|  | 43 | DS2 | 5 |  |  |  |  |  |  |  |  |  |
|  | 44 | DS2 | 8 | -1.07 | 0.30 | -0.02 | 0.17 |  |  |  |  |  |
|  | 45 | DS2 | 8 |  |  |  |  |  |  |  |  |  |
|  | 46 | DS2 | 10 | -0.87 | 0.17 | 0.04 | 0.10 |  | 0.7549 | 2238 | 2310 | 15 |
|  | 47 | DS2 | 10 |  |  |  |  |  |  |  |  |  |
|  | 48 | DS2 | 12 |  |  |  |  |  |  |  |  |  |
|  | 49 | DS2 | 12 |  |  |  |  |  |  |  |  |  |
|  | 50 | DS2 | 12 | -1.04 | 0.17 | 0.01 | 0.19 |  |  |  |  |  |
| 2019 | 1 | DS3 | 26 | -0.95 | 0.20 | 0.01 | 0.18 | 144.53 | 0.8956 | 769 | 841 | 15 |
| 2017 | 2 | DS3 | 23 |  |  |  |  | 80.77 |  |  |  |  |
| 2014 | 3 | DS3 | 25 | -0.50 | 0.25 | 0.03 | 0.13 | 71.74 | 0.7845 | 1874 | 1946 | 15 |
| 2012 | 4 | DS3 | 27 |  |  |  |  | 110.77 |  |  |  |  |
| 2011 | 5 | DS3 | 26 | -1.06 | 0.21 | -0.04 | 0.15 | 124.30 | 0.7586 | 2224 | 2296 | 15 |
| 2008 | 6 | DS3 | 24 |  |  |  |  | 96.12 |  |  |  |  |
| 2006 | 7 | DS3 | 25 | -0.87 | 0.21 | -0.01 | 0.15 | 111.32 | 0.7528 | 2322 | 2394 | 20 |
| 2005 | 8 | DS3 | 26 |  |  |  |  | 186.74 |  |  |  |  |
| 2003 | 9 | DS3 | 25 | -0.68 | 0.19 | 0.05 | 0.22 | 127.76 | 0.7538 | 2311 | 2383 | 20 |
| 2002 | 10 | DS3 | 25 |  |  |  |  | 193.30 |  |  |  |  |
| 2000 | 11 | DS3 | 25 |  |  |  |  | 95.08 |  |  |  |  |
| 1996 | 12 | DS3 | 25 | -0.88 | 0.22 | 0.01 | 0.29 | 62.26 | 0.7455 | 2357 | 2429 | 20 |
| 1993 | 13 | DS3 | 25 |  |  |  |  | 64.99 |  |  |  |  |
| 1989 | 14 | DS3 | 24 |  |  |  |  | 49.62 |  |  |  |  |
| 1984 | 15 | DS3 | 23 | -1.02 | 0.17 | -0.02 | 0.09 | 60.58 | 0.7109 | 2824 | 2896 | 30 |
| 1978 | 16 | DS3 | 22 |  |  |  |  | 30.47 |  |  |  |  |
| 1973 | 17 | DS3 | 20 |  |  |  |  | 38.74 |  |  |  |  |
| 1967 | 18 | DS3 | 20 | -0.51 | 0.18 | -0.08 | 0.18 | 34.08 | 0.7644 | 2149 | 2221 | 20 |
| 1960 | 19 | DS3 | 20 |  |  |  |  | 25.76 |  |  |  |  |
| 1950 | 20 | DS3 | 15 | -1.00 | 0.12 | 0.00 | 0.19 | 13.44 |  |  |  |  |
| 1937 | 21 | DS3 | 11 |  |  |  |  | 5.66 |  |  |  |  |
| 1922 | 22 | DS3 | 11 | -0.97 | 0.06 | -0.02 | 0.04 | 5.63 | 0.7656 | 2128 | 2200 | 20 |
| 1913 | 23 | DS3 | 11 |  |  |  |  | 12.34 |  |  |  |  |
| 1902 | 24 | DS3 | 13 | -1.40 | 0.10 | -0.06 | -0.01 | 9.87 |  |  |  |  |
|  | 25 | DS3 | 10 |  |  |  |  |  |  |  |  |  |
| 2023 | 1 | DS4 | 54 | -1.24 | 0.18 | -0.03 | 0.09 | 154.45 | 0.9473 | 502 | 574 | 20 |
| 2020 | 2 | DS4 | 50 |  |  |  |  | 77.55 |  |  |  |  |
| 2017 | 3 | DS4 | 49 | -1.21 | 0.15 | 0.02 | 0.10 | 63.69 | 0.9255 | 601 | 673 | 15 |
| 2013 | 4 | DS4 | 53 |  |  |  |  | 56.01 |  |  |  |  |
| 2005 | 5 | DS4 | 57 | -1.25 | 0.11 | -0.09 | 0.08 | 33.92 | 0.9753 | 179 | 251 | 15 |
| 2000 | 6 | DS4 | 56 |  |  |  |  | 56.73 |  |  |  |  |
| 1991 | 7 | DS4 | 52 | -1.14 | 0.14 | -0.04 | 0.11 | 23.91 | 0.9571 | 386 | 458 | 20 |
| 1980 | 8 | DS4 | 55 |  |  |  |  | 11.98 |  |  |  |  |
| 1963 | 9 | DS4 | 62 | -1.15 | 0.20 | 0.04 | 0.18 | 12.00 | 1.0011 |  | 75 | 15 |
| 1961 | 10 | DS4 | 57 |  |  |  |  | 94.02 |  |  |  |  |
| 1958 | 11 | DS4 | 58 | -1.14 | 0.13 | 0.00 | 0.11 | 112.75 | 0.9285 | 606 | 678 | 20 |
| 1957 | 12 | DS4 | 52 |  |  |  |  | 136.09 |  |  |  |  |
| 1953 | 13 | DS4 | 57 | -0.86 | 0.20 | 0.09 | 0.17 | 76.89 | 1.0035 |  | 76 | 20 |
| 1950 | 14 | DS4 | 49 |  |  |  |  | 81.40 |  |  |  |  |
| 1948 | 15 | DS4 | 48 | -1.04 | 0.10 | 0.00 | 0.04 | 167.70 | 0.9858 | 107 | 179 | 15 |
| 1946 | 16 | DS4 | 47 |  |  |  |  | 267.72 |  |  |  |  |
| 1944 | 17 | DS4 | 47 |  |  |  |  | 160.12 |  |  |  |  |
| 1943 | 18 | DS4 | 38 | -1.12 | 0.11 | -0.02 | 0.04 | 267.97 | 0.9151 | 669 | 741 | 15 |
| 1940 | 19 | DS4 | 34 |  |  |  |  | 157.73 |  |  |  |  |
| 1939 | 20 | DS4 | 30 | -0.99 | 0.08 | 0.00 | -0.02 | 192.77 | 0.7146 | 2795 | 2867 | 20 |
| 1937 | 21 | DS4 | 29 |  |  |  |  | 287.64 |  |  |  |  |
|  | 22 | DS4 | 27 | -0.83 | -0.02 | -0.08 | -0.05 | 327.43 | 0.3806 | 8542 | 8614 | 25 |
| 1937 | 23 | DS4 | 27 |  |  |  |  | 259.06 |  |  |  |  |
|  | 24 | DS4 | 26 |  |  |  |  | 239.01 |  |  |  |  |
| 1936 | 25 | DS4 | 26 | -0.85 | 0.00 | -0.04 | 0.01 | 260.59 | 0.3047 | 10930 | 11002 | 30 |
|  | 26 | DS4 | 27 |  |  |  |  | 173.32 |  |  |  |  |
| 1935 | 27 | DS4 | 27 | -1.18 | -0.04 | -0.03 | -0.12 | 245.19 |  |  |  |  |
| 1933 | 28 | DS4 | 26 |  |  |  |  | 155.01 |  |  |  |  |
|  | 29 | DS4 | 27 |  |  |  |  | 150.63 |  |  |  |  |
| 1931 | 30 | DS4 | 28 | -1.13 | 0.02 | -0.06 | 0.03 | 120.57 | 0.7585 | 2226 | 2298 | 20 |
| 1929 | 31 | DS4 | 26 |  |  |  |  | 141.72 |  |  |  |  |
| 1928 | 32 | DS4 | 26 | -1.07 | -0.09 | -0.08 | -0.09 | 117.81 |  |  |  |  |
| 1926 | 33 | DS4 | 25 |  |  |  |  | 129.73 |  |  |  |  |
|  | 34 | DS4 | 25 |  |  |  |  | 259.97 |  |  |  |  |
| 1925 | 35 | DS4 | 25 | -1.11 | -0.01 | -0.01 | -0.01 | 251.77 | 0.6462 | 3775 | 3847 | 25 |
|  | 36 | DS4 | 26 |  |  |  |  | 206.06 |  |  |  |  |
| 1923 | 37 | DS4 | 26 | -1.20 | -0.03 | -0.08 | -0.03 | 218.15 |  |  |  |  |
| 1922 | 38 | DS4 | 26 |  |  |  |  | 135.59 |  |  |  |  |
|  | 39 | DS4 | 26 |  |  |  |  | 125.54 |  |  |  |  |
| 1918 | 40 | DS4 | 26 | -0.99 | -0.08 | -0.05 | -0.13 | 114.83 |  |  |  |  |
|  | 41 | DS4 | 27 |  |  |  |  | 131.85 |  |  |  |  |
| 1915 | 42 | DS4 | 26 | -1.01 | 0.02 | -0.03 | 0.03 | 119.25 |  |  |  |  |
| 1913 | 43 | DS4 | 25 |  |  |  |  | 147.25 |  |  |  |  |
|  | 44 | DS4 | 26 |  |  |  |  | 83.00 |  |  |  |  |
| 1906 | 45 | DS4 | 28 | -1.01 | 0.05 | -0.02 | 0.02 | 103.73 | 0.4538 | 7269 | 7341 | 25 |
|  | 46 | DS4 | 26 |  |  |  |  | 105.60 |  |  |  |  |
| 1903 | 47 | DS4 | 27 | -1.06 | 0.00 | -0.05 | -0.08 | 83.29 |  |  |  |  |
| 1898 | 48 | DS4 | 26 |  |  |  |  | 43.63 |  |  |  |  |
|  | 49 | DS4 | 25 |  |  |  |  | 55.76 |  |  |  |  |
| 1891 | 50 | DS4 | 27 | -1.11 | 0.06 | -0.01 | 0.05 | 86.99 |  |  |  |  |
| 2020 | 1 | DS5 | 10 | -0.19 | 0.08 | 0.00 | 0.06 | 47.02 | 0.9318 | 596 | 667 | 54 |
| 2016 | 5 | DS5 | 11 | -1.61 | 0.30 | 0.01 | 0.22 | 70.71 |  |  |  |  |
| 2013 | 8 | DS5 | 11 | -1.61 | 0.28 | 0.00 | 0.22 | 47.10 | 0.8817 | 925 | 996 | 61 |
| 2009 | 11 | DS5 | 10 | -1.18 | 0.22 | -0.04 | 0.20 | 46.95 |  |  |  |  |
| 2006 | 14 | DS5 | 12 | -0.55 | 0.07 | 0.02 | 0.09 | 59.95 | 0.8259 | 1404 | 1475 | 78 |
| 2003 | 16 | DS5 | 11 | -0.62 | 0.04 | -0.03 | 0.01 | 59.81 |  |  |  |  |
| 2000 | 18 | DS5 | 8 | -2.17 | 0.24 | 0.02 | 0.23 | 60.18 |  |  |  |  |
| 1997 | 20 | DS5 | 12 | -2.11 | 0.29 | 0.03 | 0.14 | 49.32 | 0.8221 | 1461 | 1532 | 63 |
| 1994 | 22 | DS5 | 11 | -0.56 | 0.04 | 0.01 | 0.05 | 43.17 |  |  |  |  |
| 1988 | 25 | DS5 | 16 | -0.69 | 0.01 | 0.02 | 0.03 | 37.26 | 0.8656 | 1067 | 1138 | 90 |
| 1982 | 28 | DS5 | 17 | -1.24 | 0.19 | 0.03 | 0.16 | 40.99 |  |  |  |  |
| 1975 | 31 | DS5 | 16 | -1.49 | 0.28 | 0.03 | 0.18 | 31.21 | 0.8342 | 1337 | 1408 | 37 |
| 1963 | 36 | DS5 | 12 | -0.45 | 0.01 | -0.02 | 0.04 | 28.37 |  |  |  |  |
| 1950 | 39 | DS5 | 13 | -1.77 | 0.23 | -0.03 | 0.15 | 12.39 | 0.8227 | 1458 | 1529 | 64 |
| 1932 | 42 | DS5 | 9 | -1.58 | 0.21 | 0.03 | 0.14 | 9.81 |  |  |  |  |
| 1873 | 48 | DS5 | 11 | -1.91 | 0.19 | 0.00 | 0.16 | 3.56 | 0.8046 | 1635 | 1706 | 60 |

**Supplementary Data references**

1. A. Schirone *et al.*, Assessment of measurement accuracy in 210Pb dating sediment methods. *Quaternary Geochronology* **69** (2022).

2. H. A. Polach, M. Stuiver, Discussion Reporting of 14C Data. *Radiocarbon* **19**, 355-363 (1977).

3. X. Wang *et al.*, Using Mercury Isotopes To Understand Mercury Accumulation in the Montane Forest Floor of the Eastern Tibetan Plateau. *Environ Sci Technol* **51**, 801-809 (2017).

4. X. Wang *et al.*, Global warming accelerates uptake of atmospheric mercury in regions experiencing glacier retreat. *Proc Natl Acad Sci U S A* **117**, 2049-2055 (2020).

5. X. Wang, W. Yuan, C. J. Lin, F. Wu, X. Feng, Stable mercury isotopes stored in Masson Pinus tree rings as atmospheric mercury archives. *J Hazard Mater* **415**, 125678 (2021).

6. J. D. Blum, B. A. Bergquist, Reporting of variations in the natural isotopic composition of mercury. *Analytical and Bioanalytical Chemistry* **388**, 353-359 (2007).

7. N. Estrade, J. Carignan, J. E. Sonke, O. F. X. Donard, Measuring Hg Isotopes in Bio-Geo-Environmental Reference Materials. *Geostand Geoanal Res* **34**, 79-93 (2010).

8. X. Wang, W. Yuan, C.-J. Lin, X. Feng, Mercury cycling and isotopic fractionation in global forests. *Crit Rev Env Sci Tec* **52**, 3763-3786 (2021).

9. R. Sun *et al.*, Modelling the mercury stable isotope distribution of Earth surface reservoirs: Implications for global Hg cycling. *Geochimica et Cosmochimica Acta* **246**, 156-173 (2019).

10. X. Wang *et al.*, Underestimated Sink of Atmospheric Mercury in a Deglaciated Forest Chronosequence. *Environ Sci Technol* **54**, 8083-8093 (2020).

11. N. Liu *et al.*, Quantifying Mercury Distribution and Source Contribution in Surface Soil of Qinghai-Tibetan Plateau Using Mercury Isotopes. *Environ Sci Technol* **57**, 5903-5912 (2023).

12. J. J. Zhu, M. Yang, Z. J. Ren, Machine Learning in Environmental Research: Common Pitfalls and Best Practices. *Environ Sci Technol* 10.1021/acs.est.3c00026 (2023).

13. X. Wang *et al.*, Root uptake dominates mercury accumulation in permafrost plants of Qinghai-Tibet Plateau. *Communications Earth & Environment* **3** (2022).
